# Prosthetic Valve *Candida* Endocarditis: A Case Report with 18F-FDG-PET/CT as Part of the Diagnostic Workup

**DOI:** 10.1155/2020/4921380

**Published:** 2020-11-26

**Authors:** Soile Pauliina Salomäki, Antti Saraste, Päivi Jalava-Karvinen, Laura Pirilä, Ulla Hohenthal

**Affiliations:** ^1^Department of infectious Diseases, Division of Medicine, Turku University Hospital, Turku, Finland; ^2^Faculty of Medicine, Department of Clinical Medicine, University of Turku, Turku, Finland; ^3^Turku PET Centre, University of Turku and Turku University Hospital, Turku, Finland; ^4^Heart Center, Turku University Hospital, Turku, Finland; ^5^Department of Rheumatology, Division of Medicine, Turku University Hospital, Turku, Finland

## Abstract

Diagnosis of *Candida* spp. infective endocarditis (IE) is challenging, and diagnostic delays are common. We describe two patients with *Candida* spp. prosthetic valve endocarditis (PVE) and ^18^fluorodeoxyglucose positron emission tomography/computed tomography (^18^F-FDG-PET/CT) as a part of diagnostic workup. We also refer to 5 other cases we found from the published literature. These cases highlight that ^18^F-FDG-PET/CT can improve diagnostic accuracy in prosthetic valve *Candida* endocarditis.

## 1. Background

*Candida* spp. endocarditis is a rare but devastating disease accounting for <2% of all IE and ~3% of prosthetic valve endocarditis (PVE) [1]. Candida endocarditis is associated with up to 36% in hospital mortality and up to 59% 1-year mortality [2, 3]. Because of the inconsistent and nonspecific presentation, the diagnosis of candida endocarditis is often delayed. Previous studies have shown ^18^F-FDG PET/CT as a valuable tool in the diagnosis of bacterial PVE but less is known about its role in *Candida* endocarditis [4–7].

We describe 2 cases of prosthetic valve *Candida* endocarditis in which a whole-body or cardiac ^18^F-FDG-PET/CT scan (Discovery VCT, General Electric Medical Systems, Milwaukee, WI, USA) was used in the diagnostic workup of the disease. Patients were on low-carbohydrate diet for 24 hours before the PET-scan and fasted at least 10 hours before the study.

## 2. Case Presentations

### 2.1. Case 1

**Figure 1:**
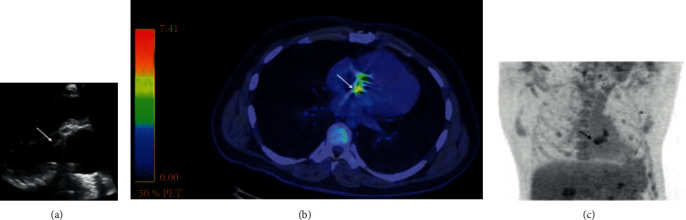
Transesophageal echocardiography image of patient 1 (panel (a)); arrow shows vegetation. ^18^F-FDG-PET/CT images of patient 1 (panels (b) and (c)). Arrows show increased ^18^F-FDG uptake at the site of aortic valve prosthesis.

A 39-year-old man with a history of intravenous drug use (IVDU) and chronic hepatitis C infection presented to our university hospital due to a two-week history of fever, 4-day history of low back pain, and 1-day history of problems in keeping his balance. He had a history of aortic valve replacement with a mechanical prosthesis due to IE 8 years earlier. On admission, the physical examination was remarkable for systolic murmur on auscultation, multiple peripheral emboli of lower limbs, and clumsiness of the right upper limb. His temperature was 37.9°C, oxygen saturation 88%, heart rate 103 beats per minute, and blood pressure 141/81 mmHg. The C-reactive protein (CRP) was 189 mg/L, white blood cells (WBC) 15.8 × 10^9^/L, hemoglobin (Hb) level 106 g/L, and creatinine 89 *μ*mol/L. Transesophageal echocardiography (TEE) confirmed the vegetation of 9 × 16 mm (Figure 1) detected at admission with transthoracic echocardiography (TTE). Magnetic resonance imaging (MRI) of the head revealed multiple cerebral hemorrhagic lesions and an abscess in the cerebellum. Embolic infarctions were detected in the spleen and in both kidneys by computed tomography (CT). Blood cultures taken on admission yielded *Candida albicans* on day 4, and therapy with liposomal amphotericin B (LAMB) was started. ^18^F-FDG-PET/CT of the thoracic region was performed on day 19. Injected dose of ^18^F-FDG was 392 MBq and 47 minutes later, a whole-body PET acquisition (3 min per bed position) was performed following low-dose CT scan without contrast. High focal uptake was detected at the site of the mechanic aortic valve with a maximal standardized uptake value (SUV_max_) of 7.4 g/mL (Figure 1). The patient died on day 30 after admission due to massive intracerebral hematoma.

### 2.2. Case 2

**Figure 2:**
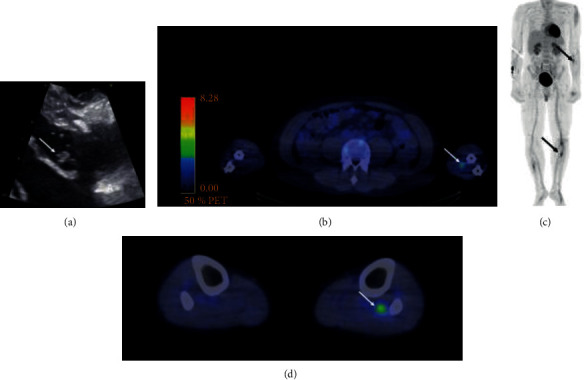
Transesophageal echocardiography images of patient 2 (panel (a)). Arrow shows vegetation. ^18^F-FDG-PET/CT images of patient 2 (panels (b)–(d)). Arrows show ^18^F-FDG uptake at the site brachial artery and fibular artery.

A 31-year-old male with previous IVDU was admitted to our hospital for fever and pain in the left hand and right leg. He had chronic hepatitis C infection and a history of aortic valve replacement with mechanical prosthesis due to IE 4 years earlier. On admission, his temperature was 38.1°C, oxygen saturation 97%, heart rate 94 beats per minute, and blood pressure 140/80 mmHg. On the physical examination, no pulsation was identified in radial or ulnar arteries of the left wrist but the hand was warm. Blood tests showed WBC count of 8.8 × 10^9^/L, Hb 137 g/L, CRP 151 mg/L, and creatinine 82 *μ*mol/L. Blood cultures remained negative. No signs of endocarditis was detected on TTE. However, ^18^F-FDG-PET/CT (injected dose of ^18^F-FDG 278 MBq, time from injection to scan 50 min following low-dose CT without contrast) revealed focal uptake consistent with embolic foci in the brachial artery and in arteries of both legs (Figure 2). No accumulation of ^18^FDG was detected on the prosthetic valve but due to the noncompliance of the patient with the diet before PET/CT, there was physiological uptake in myocardium. CT angiography showed mycotic aneurysm in the brachial artery and thrombosis of the left popliteal artery. The patient was treated with vancomycin, gentamycin, and rifampicin for IE. The patient had intermittently fever, and inflammation values were persistently high, CRP at maximum 322 mg/L and ESR 89 mm/h. Blood cultures were negative until blood culture taken on day 21 after admission yielded *C. albicans*, and treatment with micafungin was commenced. Vegetation on the prosthetic valve was later found on TEE (Figure 2). On day 22. the patient suffered from massive infarct of the right hemisphere followed by hemicraniectomy. The patient remained in poor clinical condition and died 6 months later.

## 3. Discussion

^18^FDG-PET/CT has emerged as a valuable diagnostic tool for IE in cases with diagnostic difficulties especially in PVE. There is increasing evidence that besides localizing bacterial infections,^18^FDG-PET/CT can also recognize sites of fungal infections [8]. However, less is known about the diagnostic yield of ^18^F-FDG-PET/CT in fungal PVE. Because of the rarity of the disease, it is challenging to carry out a prospective clinical trial.

To our knowledge, only 5 cases have previously been published in which PET/CT has been used in diagnostic workup of PVE caused by *Candida* sp. [4–6, 9]. As in one of our cases, focal uptake was recognized on the prosthetic valve in all of these 5 previous cases (Table 1). In the other case of ours, no uptake was found on the prosthetic valve. Instead, embolic findings consistent with endocarditis were detected by PET/CT. One explanation for the negative finding of the valve area on PET/CT might be the biofilm formation on prosthesis which is typical for *Candida* species and enables *Candida* to evade the host immune response [10].

One can also question the diagnosis of the etiological agent in our case with the first positive blood culture on day 21 after admission as *C. albicans* might also present secondary infection. However, no other etiological agent was identified, no response was achieved with antibiotic treatment, and course of the illness with multiple emboli was typical for *Candida*. Moreover, the sensitivity of the blood culture to detect *Candida* has been estimated to be only 50-75% [11]. As in our case, the negative blood culture may be one of the reasons for the delayed diagnosis of the disease which is typical for fungal endocarditis.

Embolic complications have been found in up to 46% of the patients with *Candida* endocarditis [3]. As in our case, these can be detected by the whole-body ^18^FDG-PET/CT which enables detection of several embolic findings with one diagnostic modality and as in our case, these findings on PET/CT may confirm the diagnosis of endocarditis. The multiple arterial findings could have also arisen the suspicion of *Candida* as an etiological agent. Unfortunately, in the other case with multiple emboli, the PET/CT was restricted only to thorax, and we were not able to evaluate the usefulness of PET/CT to detect peripheral embolic foci in this case.

Our 2 cases and one of the previously published cases are case reports. Consequently, findings based on these 7 cases must be interpreted with caution taking into account the possible selection bias of reported cases.

In conclusion, our two cases and the previously published cases indicate that ^18^F-FDG-PET/CT seems to be a promising and valuable diagnostic tool in *Candida* spp. prosthetic valve endocarditis.

## Figures and Tables

**Table 1:** The characteristics of 7 patients with *Candida* spp. prosthetic valve endocarditis.

Patient [reference number]	*Candida* species	Type and location of prosthetic valve	Transesophageal echocardiography	Visual assessment of valves by FDG-PET/CT	Duke criteria
1 [4]	*C. parapsilosis*	Mechanical AV and MV	Negative^a^	Positive	Definitive
2 [4]	*C. parapsilosis*	Mechanical AV	Fistula^a^	Positive	Definitive
3 [5]	*Candida* ^b^	ND^b^	ND	Positive	Definitive
4 [6]	*C. albicans*	AV^c^	Increased wall thickness of the aortic root	Positive	Definitive
5 [9]	*C. albicans*	Mechanical AV	Negative	Positive	Definitive
6 [present case 1]	*C. albicans*	Mechanical AV	Vegetation	Positive	Definitive
7 [present case 2]	*C. albicans*	Mechanical AV	Vegetation	Negative^d^	Definitive

Abbreviations: AV: aortic valve; MV: mitral valve; ND: not done or data not given; ^a^Transthoracic echocardiography. ^b^Species not given. ^c^Type of the prosthetic valve not given. ^d^ Embolic foci in peripheral arteries detected by^18^F-FDG-PET/CT.
